# Cox1 mutation abrogates need for Cox23 in cytochrome *c* oxidase biogenesis

**DOI:** 10.15698/mic2016.07.511

**Published:** 2016-06-30

**Authors:** Richard Dela Cruz, Mi-Young Jeong, Dennis R. Winge

**Affiliations:** 1 University of Utah Health Sciences Center, Departments of Medicine and Biochemistry, Salt Lake City, Utah 84132, USA.; 2 Present address: Oklahoma Medical Research Foundation, Oklahoma City, OK 73104, USA.

**Keywords:** cytochrome oxidase, mitochondria, COX1, COX23

## Abstract

Cox23 is a known conserved assembly factor for cytochrome *c* oxidase, although its role in cytochrome *c* oxidase (CcO) biogenesis remains unresolved. To gain additional insights into its role, we isolated spontaneous suppressors of the respiratory growth defect in *cox23*∆ yeast cells. We recovered independent colonies that propagated on glycerol/lactate medium for *cox23*∆ cells at 37°C. We mapped these mutations to the mitochondrial genome and specifically to *COX1* yielding an I^101^F substitution. The I^101^F Cox1 allele is a gain-of-function mutation enabling yeast to respire in the absence of Cox23. CcO subunit steady-state levels were restored with the I^101^F Cox1 suppressor mutation and oxygen consumption and CcO activity were likewise restored. Cells harboring the mitochondrial genome encoding I^101^F Cox1 were used to delete genes for other CcO assembly factors to test the specificity of the Cox1 mutation as a suppressor of *cox23*∆ cells. The Cox1 mutant allele fails to support respiratory growth in yeast lacking Cox17, Cox19, Coa1, Coa2, Cox14 or Shy1, demonstrating its specific suppressor activity for *cox23*∆ cells.

## INTRODUCTION

Cytochrome *c* oxidase (CcO) of the mitochondrial respiratory chain couples the reduction of molecular oxygen with proton translocation across the inner membrane (IM) to generate the membrane potential used to synthesize ATP. Mammalian CcO contains 14 subunits in which the 3 core subunits (Cox1-Cox3) are encoded by the mitochondrial genome 1,2. This catalytic core is surrounded by nuclear-encoded subunits, which confer enzyme stability and provide sites for the regulation of its activity 3,4. The fully assembled holoenzyme is further organized into supercomplexes with other respiratory complexes 5,6,7,8. Subunit 1 (Cox1) of CcO contains two heme *a* and one copper (Cu) ion as cofactors 9. One heme *a* functions in electron transfer, whereas the second heme *a* (heme *a*_3_) has an open coordinate site where O_2 _binds in a reaction center that also contains the Cu_B_ site. Cox2 contains a cysteine-bridged, binuclear Cu site (Cu_A_) within a soluble globular domain that serves as the site of electron transfer from reduced cytochrome *c*.

The assembly of CcO requires a myriad of steps including the coordinated assembly of subunits translated on cytoplasmic and mitochondrial ribosomes and insertion of heme *a* and copper cofactors. Studies with yeast mutants impaired in heme *a* biosynthesis and CcO biogenesis have revealed that CcO assembly proceeds in a modular fashion with Cox1 maturation preceding independently of Cox2 or Cox3 maturation 10,11,12**.** Over 40 yeast accessory proteins have been found to be important for the assembly of CcO 13,14,15.

Hemylation and copper ion insertion are processes that occur within the intermembrane space (IMS) of mitochondria. The final step in heme *a* formation is catalyzed by Cox15, which has its catalytic domain projecting into the IMS. The mechanism of insertion of heme *a* into Cox1 is not resolved, but this process is assisted by the IM protein Shy1 16. Copper ion metallation of Cox1 and Cox2 initiates within the IMS by the Cu(I) donor protein Cox17 17. Cox17-mediated Cu(I) donation involves two accessory factors Cox11 and Sco1 that function in the metallation of the Cu_B_ site in Cox1 and Cu_A_ site in Cox2, respectively 18,19. Both Cox11 and Sco1 are inner membrane (IM)-associated proteins with Cu(I)-binding globular domains protruding into the IMS. Cox17-mediated Cu(I) transfer to Sco1 is followed by the subsequent transfer to Cox2 in a reaction dependent on a key redox role of Sco2 in metazoans 19,20. Likewise, Cox17-mediated Cu(I) transfer to Cox11 is believed to occur prior to transfer to Cox1 forming the Cu_B_ center 18,21,22.

Cox17 is part of a family of IMS proteins including CcO assembly factors Cox19, Cox23, Pet191 and Cmc1 that all possess a conserved twin Cx_9_C structural motif 17,23,24. Cox17 forms a helical hairpin conformation stabilized by two disulfide bonds of the twin Cx_9_C cysteines 25,26,27. Cox17 has 2 additional conserved Cys residues upstream of the first Cys of the twin Cx_9_C motif, and these vicinal thiolates bind Cu(I) in a bis-coordinate complex 27. A second of these twin Cx_9_C proteins Cox19 lacks the additional Cu(I) binding residues of Cox17 and was recently shown to interact with the inner membrane Cox11 protein and mediates the redox regulation of Cox11 28.

Cox23 also lacks the Cu-binding Cys residues and is not expected to bind Cu(I) *in vivo*. Yeast lacking Cox23 are CcO-deficient but residual levels of the enzyme persist 29. The respiratory defect in *cox23*∆ cells is partially suppressed by overexpression of Cox17 in cells only when cultured in 2 mM CuSO_4 _29. This observation led to speculation that Cox23 functions in Cu delivery to CcO during its biogenesis. This prediction is consistent with a recent study in human cells. The abundance of the human Cox23 ortholog is attenuated in fibroblasts or myoblasts isolated from patients with mutations in SCO1 or SCO2 30. Furthermore, the abundance of Cox23 was attenuated in control fibroblasts treated with a Cu chelator to deplete cellular copper 30.

In the absence of any clear functional data on Cox23, we screened for spontaneous suppressors of the respiratory defect of *cox23*∆ cells. In this report, we describe the isolation of a robust suppressor of the respiratory defect in *cox23*∆ cells that mapped to the mitochondrial-encoded Cox1 subunit.

## RESULTS AND DISCUSSION

**Figure 1:**
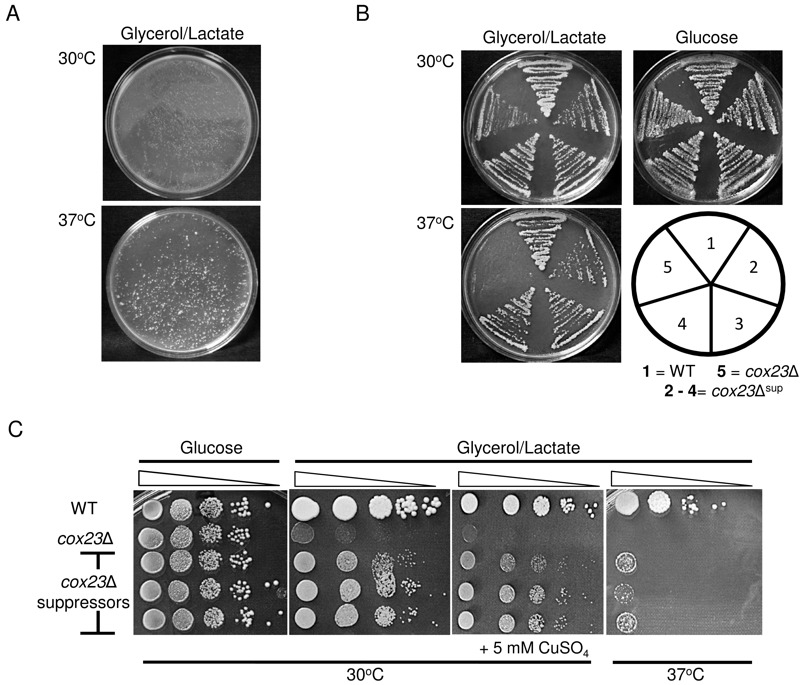
FIGURE 1: Growth properties of *cox23*Δ suppressor. **(A)** Diploid *cox23*Δ yeast cells were plated at a density of ~10^7^ cells/plate on YP glycerol/lactate (YPGL). The plates were photographed after 8 days of incubation at 30 and 37°C. **(B)** BY4743, *cox23*Δ, and *cox23*Δ suppressor cells were streaked on YPGL or YP-glucose (YPD) and incubated at the indicated temperatures. **(C)** Serial dilutions of BY4743, *cox23*Δ, and *cox23*Δ suppressors spotted on YPD or YPGL plates and incubated at 30°C for 2 and 5 days and at 37°C for 7 days, respectively (left most lane is OD_600_ = 0.5).

Yeast lacking Cox23 exhibit a partial growth defect on glycerol/lactate growth medium. The respiratory growth defect is more pronounced in BY4741 cells relative to the W303 genetic background 29. The growth impairment was sufficiently strong in BY4741 and BY4743 cells cultured at 37°C to permit a suppressor screen (Fig. 1C). We plated haploid and diploid *cox23*∆ cells (BY4741 and BY4743, respectively) at a density of ~10^7^ cells per plate on glycerol/lactate medium at both 30 and 37°C. After 8 days a series of colonies appeared initially in the diploid null cells at 37°C relative to 30°C cultures (Fig. 1A). A series of respiratory competent colonies were collected and replated under respiratory conditions. Whereas the parent diploid *cox23*∆ cells failed to propagate at 37°C, the isolated colonies retained their ability to propagate at both 30 and 37°C temperatures (Fig. 1B). In serial dilution drop tests, the suppressors grew more robustly at 30°C, but growth at 37°C although apparent but reduced relative to WT cells (Fig. 1C). The addition of exogenous copper sulfate did not enhance respiratory growth (Fig. 1C), and the addition of the copper (I) chelator bathocuproine sulfonate (25 μM) impaired *cox23*∆ cells and the *cox23*∆ suppressor strain (data not shown).

**Figure 2:**
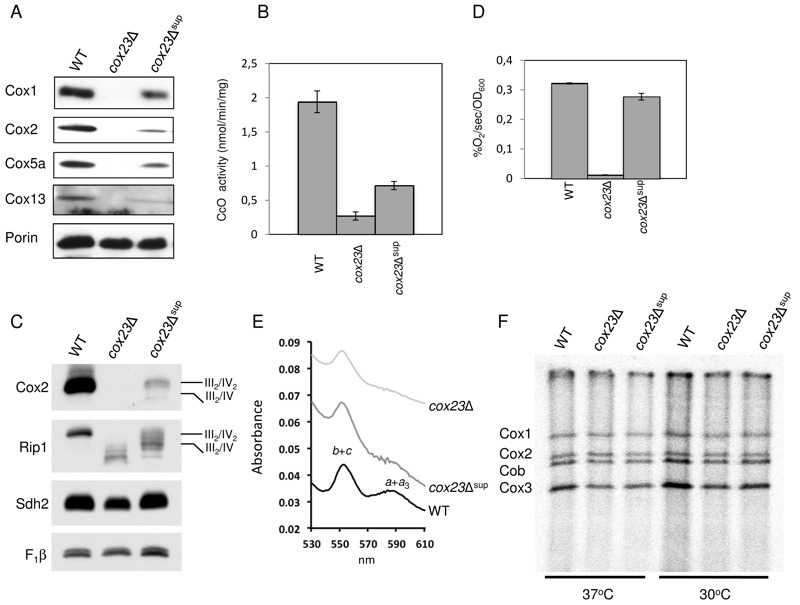
FIGURE 2: Functional characterization of *cox23*Δ suppressor. **(A)** Steady-state concentration of representative Complex IV (CIV) subunits. Total mitochondrial protein (20 μg) were separated on 12% SDS-PAGE, transferred to nitrocellulose and probed with CIV subunit specific antibodies and porin as loading control. **(B) **Mitochondria from WT, *cox23*Δ, and *cox23*Δ suppressor cells grown in liquid YPGal were purified and assayed for CcO activity (nmol cytochrome *c* oxidized/min/mg protein). The data represents the average of four independent experiments (error bars indicate standard deviation). **(C)** Total mitochondrial protein (30 μg) were solubilized in 1% digitonin, the mitochondrial protein complexes separated on a blue-native gel, then transferred to PVDF. The complexes were visualized using antibodies specific to subunits of the respiratory complexes. **(D)** WT, *cox23*Δ, and *cox23*Δ suppressor cells were grown in liquid YP-Galactose (YPGal) at 30°C overnight and carbon-swapped to YPGL for 10 hours before oxygen consumption was measured. The data represents the average of three independent experiments (error bars indicate standard deviation). 1000 cells were plated in YPD-agar to confirm viability (data not shown). **(E)** Pyridine hemochromes analysis of mitochondrial hemes. Optical absorbance spectra were recorded of reduced minus oxidized cytochromes in the shown strains. **(F)** Mitochondrial proteins of wild-type (WT), *cox23*Δ, and *cox23*Δ suppressor cells were pulse-labeled with [^35^S]-methionine for 7.5 min at 30°C and 37°C. Total protein were extracted and separated on 15% polyacrylamide gel, then dried and exposed to x-ray film.

Mitochondria isolated from the suppressor colonies grown in galactose medium at 30°C were used for evaluation of CcO function. As can be seen in Fig. 2A, mitochondria isolated from the suppressors showed a stabilization of Cox1, Cox2, Cox5a and Cox13 subunit levels relative to the diploid null cells. Consistent with the respiratory growth shown in Fig. 1, the restoration of CcO subunits was partial relative to WT cells. Consistent with the enhanced levels of CcO subunits, CcO enzymatic activity was elevated in the *cox23*∆ suppressors (Fig. 2B) and respiratory supercomplexes consisting of *bc*_1_ and CcO were increased in abundance relative to the parent *cox23*∆ null mutant (Fig. 2C). Cellular oxygen consumption was largely restored in the *cox23*∆ suppressors (Fig. 2D) and heme *a*/*a*_3_ levels were partially restored (Fig. 2E).

Mutant cells harboring the suppressor did not exhibit elevated levels of newly translated Cox1, Cox2 or Cox3 as seen in ^35^S-methionine labeling in a mitochondrial translation assay. Mitochondrial translation was equivalent between *cox23*∆ mutant cells and the suppressor strain at both 30°C and 37°C (Fig. 2F).

**Figure 3:**
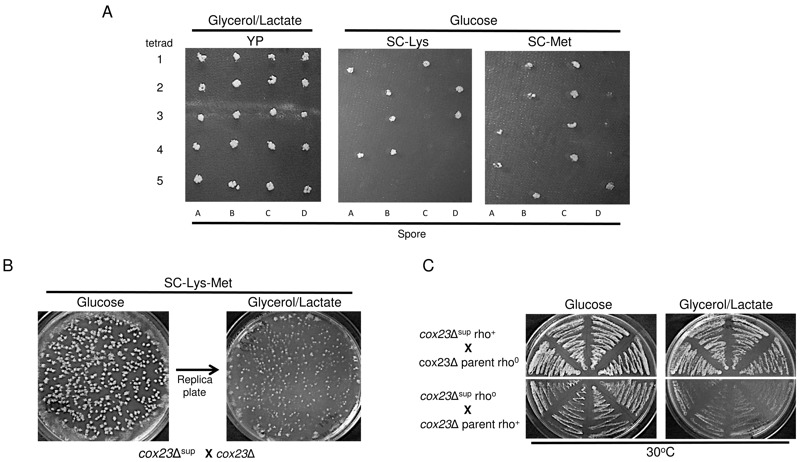
FIGURE 3:Confirmation of mtDNA origin of *cox23*Δ suppressor. **(A) **BY4743 *cox23*Δ suppressors were grown in potassium acetate solution at room temperature to induce tetrad formation. After 4-5 days, tetrads were dissected and the spores were allowed to grow on YPD-Agar. The spores were replica plated into YPGL or SCD *minus* Lys or Met Agar plates to localize the suppressor DNA. **(B)** *cox23*Δ suppressor spore was mated with *cox23*Δ haploid of the opposite mating type. Approximately 1000 cells were allowed to grow in SCD *minus *Lys and Met Agar plate. The diploid colonies were then replica plated into SCGL *minus *Lys and Met plate to confirm the mitochondrial location of the suppressor. **(C)** *cox23*Δ suppressor spore and *cox23*Δ haploid cells were exposed to EtBr to induce rho^o^ status before mating with rho^+ ^*cox23*Δ and *cox23*Δ suppressor cells of the opposite mating type, respectively. Four colonies from each mating plate were streaked on YPD and then replica plated in YPGL to confirm the *cox23*Δ mitochondrial suppressor.

Since the suppressors arose from the parent diploid BY4743 *cox23*∆ strain, the suppressor mutation was likely either due to a dominant mutation or mitochondrial DNA mutation. To distinguish between these two scenarios, we conducted tetrad dissection of the diploid suppressors (Fig. 3A). Five tetrads shown exhibited the usual 2:2 segregation of *LYS2* and *MET15* markers in the diploid strain suggesting the tetrad dissection proceeded normally. However, all four spores in each suppressor were able to grow in glycerol/lactate medium. If a dominant nuclear mutation were responsible for the suppressor phenotype, then only two of the four spores would be expected to respire. Thus, we suspected the suppressor mutation was a mitochondrial DNA mutation. To test for this, we crossed a haploid *cox23*∆ suppressor clone to a haploid *cox23*∆ clone, which is derived from tetrad dissection of *cox23*∆ suppressor, to generate diploids. The diploids were plated on either glucose or glycerol/lactate medium. As can be seen in Fig. 3B, approximately 50% of the diploids were respiratory competent in being able to propagate on glycerol/lactate medium. This is expected if the suppressor mutation was mitochondrial, since the diploids would retain either mitochondrial genome of the starting haploids.

The final proof of the mitochondrial origin of the *cox23*∆ suppressor was in generating diploids once again with a haploid *cox23*∆ suppressor clone and a haploid *cox23*∆ parent null clone, but this time starting with one of these two strains as a rho^o^ variant. The rho^o^ variants were generated by propagating the strain on ethidium bromide (EtBr) prior to conducting the cross. The resulting diploids were plated on glucose and glycerol/lactate. The only diploids capable of respiratory growth were those in which the mitochondrial DNA originated from the *cox23*∆ suppressor (Fig. 3C). The diploids obtaining mitochondrial DNA from the parental *cox23*∆ null failed to show growth on glycerol/lactate medium. This confirms that the mutation allowing respiratory growth of *cox23*∆ cells resides within the mitochondrial genome.

**Figure 4:**
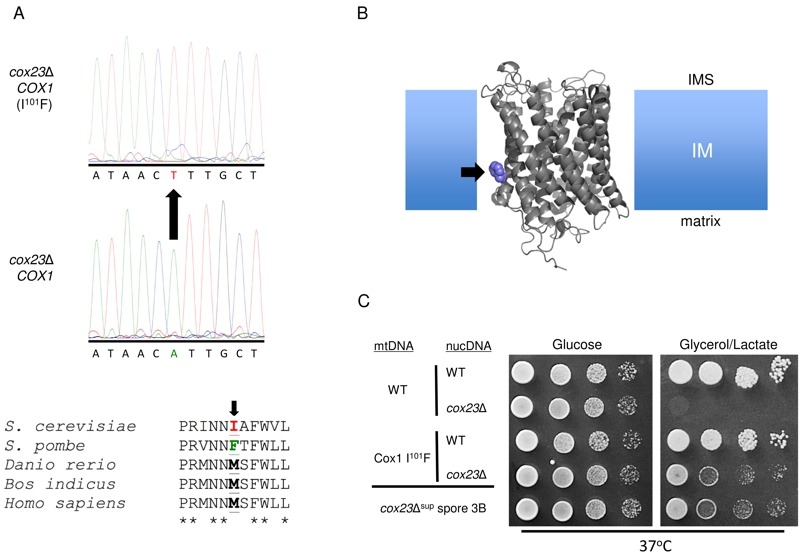
FIGURE 4: Identification of Cox1 I^101^F as the *cox23*Δ suppressor. **(A)** Cox1 ORF cDNA was isolated by RT-PCR. Sanger sequencing led to the identification of an A → T mutation causing an Ile to Phe substitution in Cox1 at position 101 that curiously is wild-type in *S. pombe* (which lacks *COX23* in its genome). **(B)** Probable location of the Cox1 mutation in the inner mitochondrial membrane based on sequence alignment to the *Bos taurus *crystal structure (1OCC.pdb). **(C)** Drop test on YPD and YPGL of Cox1 WT or I^101^F-bearing haploid cells with or without introduced *cox23* deletion (rows 1-4) compared to the *cox23*Δ spore known to contain Cox1 I^101^F (bottom, row 5).

To identify the mitochondrial mutation, DNA sequencing was carried out on *COX1*, *COX2* and *COX3 *as the most likely candidates. No mutations were identified in *COX2* or *COX3*. In contrast, an A>T mutation was identified in codon 101 of *COX1*, which leads to an Ile to Phe substitution (Fig. 4A). This is a conserved residue position in Cox1 with either Ile or Met as the common residue. A Phe is found at this corresponding position in *Schizosaccharomyces pombe* and curiously this organism lacks Cox23 in its proteome. The position of this Met in the bovine Cox1 structure lies at the start of the third transmembrane helix and projects outward toward the interface of Cox1 and Cox3 near the matrix side of inner membrane (Fig. 4B).

To confirm that the Ile^101^Phe substitution was responsible for conferring respiratory growth in *cox23*∆ cells, we isolated haploid cells containing the mutant mitochondrial genome encoding Cox1 I^101^F and subsequently deleted the *COX23* locus (Fig. 4C). Cox1 I^101^F containing yeast were competent to propagate on glycerol/lactate medium at 37°C regardless of the presence of Cox23. The respiratory growth of the *cox23*∆ cells was similar to that of the starting *cox23*∆ suppressor cells. 

**Figure 5:**
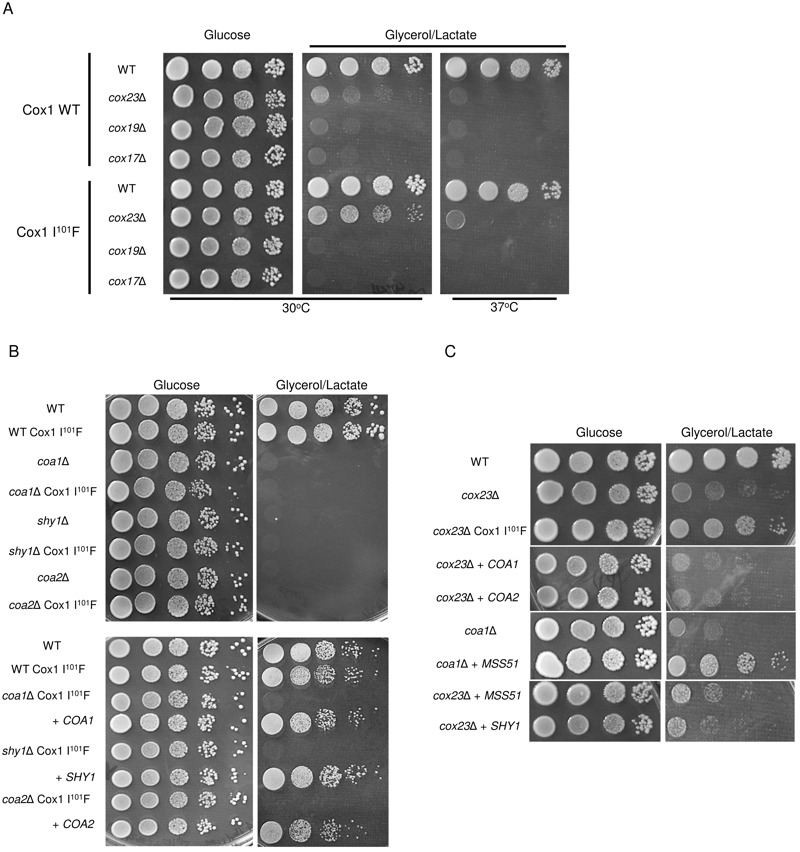
FIGURE 5: Suppression by Cox1 I^101^F is restricted to *cox23*Δ cells. **(A)** *cox17*Δ or* cox19*Δ was introduced to BY haploid wild-type nuclear DNA with or without Cox1 I^101^F mtDNA mutation and plated on YPD and YPGL plates for 3 and 7 days at 30°C respectively. **(B)** *coa1*Δ, c*oa2*Δ or* shy1*Δ was introduced to BY haploid wild-type nuclear DNA with Cox1 I^101^F mtDNA mutation and plated on YPD and YPGL plates and incubated at 30°C for 3 and 7 days respectively. **(C)** *cox23*Δ cells were transformed with *MSS51*, *SHY1*, *COA1* or *COA2* overexpression plasmids and plated on YPD and YPGL plates and incubated for 3 and 7 days at 30°C respectively.

We tested whether the Cox1 I^101^F substitution was a gain-of-function mutation specific for only *cox23*∆ cells. Cells with either a WT mitochondrial genome or the mutant *COX1* genome were used to generate deletions in CcO biogenesis genes related to *COX23*. As mentioned, Cox23 is one of several soluble twin Cx_9_C proteins present within the IMS compartment, the other two being Cox17 and Cox19. Yeast harboring deletions in either *COX17* or *COX19* failed to propagate on glycerol/lactate medium regardless of whether Cox1 had the I^101^F substitution or not (Fig. 5A). We also tested whether Cox1 I^101^F will facilitate respiratory growth in other CcO assembly mutants including *SHY1*, *COA1* and *COA2*. Yeast lacking Shy1, Coa1 or Coa2 are partially impaired in CcO biosynthesis and exhibit a respiratory defect (Fig. 5B). The presence of the mutant *COX1* allele failed to restore respiratory growth in any of these mutants, although growth was restored by vector-encoded *SHY1*, *COA1* or *COA2* in their respective mutants (Fig. 5B, bottom panel). 

Respiratory growth of certain CcO assembly mutants, e.g. *shy1*∆ and *coa1*∆ cells, can be partially restored by overexpression of the Cox1 translational activator Mss51 31**.** We tested whether *cox23*∆ cells are competent in respiratory growth upon overexpression of Mss51. Neither high levels of Mss51, Shy1, Coa1 or Coa2 were able to mediate enhanced growth, unlike the suppressor effect seen with elevated levels of Mss51 in *coa1*∆ cells (Fig. 5C). Thus, Cox1 I^101^F is a specific suppressor of *cox23*∆ cells.

**Figure 6:**
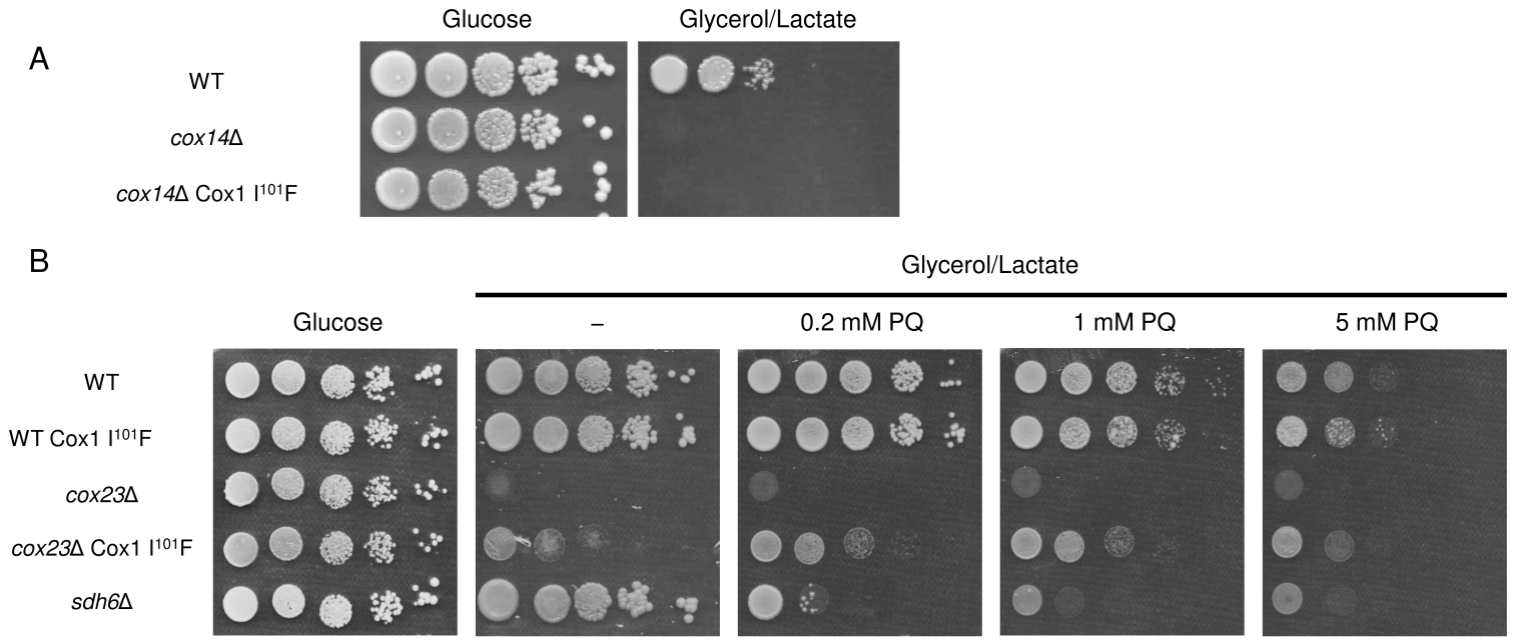
FIGURE 6: Cox1 I^101^F is unable to restore respiratory growth of *cox14*∆ cells. **(A) ***COX14* was either deleted in a WT strain or in a *cox23*∆ suppressor strain. High-copy or low-copy plasmids containing *COX23* were transformed to *cox14*∆ *cox23*∆ deletion strain to make the strain a *cox14*∆ containing C*OX1* suppressor mutation. The resulting cells were tested for respiratory growth. **(B) ***cox23*∆ mutant cells with and without the Cox1 I^101^F allele were plated on glycerol/lactate medium containing increasing concentrations of paraquat (0 - 5 mM) at 30°C.

Since *S. pombe* contains a Cox1 with a Phe at the corresponding sequence position as the Ile101 in yeast Cox1 and also lacks Cox14, we asked whether yeast containing the Cox1 I^101^F allele would be competent to respire in the absence of Cox14. To test this we introduced *COX14* deletion in *cox23*∆ suppressor strain and put plasmids-borne *COX23* back to the strain. Yeast lacking Cox14 but containing mitochondria with the Cox1 I^101^F allele were unable to propagate on glycerol/lactate rich medium (Fig. 6A). Overexpression of Cox23 from either a low or high copy vector failed to restore respiratory growth of *cox14*∆ cells containing the Cox1 Phe101 mitochondrial protein. 

Since we recovered the *cox23*∆ suppressor under respiratory condition at 37°C in which more reactive oxygen species are generated, we tested how the suppressor behave under the reactive oxidative stress condition. Yeast harboring the Cox1 I^101^F allele did not exhibit any enhanced sensitivity or resistance to reactive oxygen stress induced by culturing the cells in increasing levels of paraquat (Fig. 6B), whereas the presence of Cox1 I^101^F in *cox23*∆ cells led to a slight resistance toward paraquat. This contrasts with the known paraquat sensitivity of *sdh6*∆ cells 32. 

In summary, we recovered independent colonies that propagated on glycerol/lactate medium for *cox23*∆ cells. We mapped these mutations to the mitochondrial genome and specifically to *COX1* yielding an I^101^F substitution. The I^101^F Cox1 allele is a gain-of-function mutation enabling yeast to respire in the absence of Cox23. CcO steady-state levels were restored with the I^101^F Cox1 suppressor mutation and oxygen consumption and CcO activity were likewise restored. The allele fails to support respiratory growth in yeast lacking Cox17, Cox19 Coa1, Coa2, Cox14 or Shy1, demonstrating its specific suppressor activity for *cox23*∆ cells. 

Most species have an Ile, Leu or Met at the corresponding Ile101 sequence position. In contrast, *S. pombe* has a Phe at this position in Cox1. The gain-of-function I^101^F Cox1 allele in the *S. cerevisiae cox23*Δ deletion mutant may provide an explanation for the lack of Cox23 in *S. pombe*. Ile101 is situated at the start of TM3 in Cox1 near the matrix side of the IM. This residue in the bovine CcO structure projects outward packing against the first TM helix in Cox3 33 (Fig. 4B). 

Despite the present studies, the function of Cox23 in CcO biogenesis remains unresolved. This work reveals a potential role in Cox1 maturation. Recently, the Cox23 homolog Cox19 was shown to shield the Cu_B_ metallochaperone Cox11 from oxidation on a membrane proximal cysteinyl residue 28. If Cox23 has a related function, the redox state of the remaining two Cu(I)-binding cysteinyl residues in Cox11 may be dependent on Cox23. Cox19 and Cox23 may have non-redundant roles with Cox11 in forming the Cox1 Cu_B_ site in many species, but the I^101^F Cox1 allele may permit Cox19 to perform both functions in *S. pombe* and the present *S. cerevisiae *mutant. Alternatively, Cox23 may have a novel role in the hemylation of Cox1 in an unresolved heme *a* transfer step. Further research is needed to resolve these scenarios.

## MATERIALS AND METHODS

### Yeast Strains and Vectors

The *Saccharomyces cerevisiae* yeast strains used in this study were from a yeast knockout collection (Invitrogen). The *COA1*, *COA2*, *SHY1*, and *MSS51* ORFs were cloned into plasmid pRS413 and pRS416 under control of the *MET25* promoter and *CYC1* terminator. Yeast strains were transformed using lithium acetate. Yeast cells were cultured either in rich medium (YP) or synthetic complete (SC) medium lacking the appropriate nutrients for plasmid selection. Final concentration of carbon sources used (glucose, galactose, glycerol, lactate) in liquid media and agar plates is 2% except for raffinose (0.2%). Spores from diploid transformants were recovered after sporulation in 0.3% potassium acetate for 5 days at room temperature (RT). Rho^o^ cells were obtained after overnight incubation of yeast cells in YP-Glucose (YPD) with ethidium bromide (EtBr) at RT. 

**Table 1:** Genotype and sources of yeast strains.

**Strain**	**Genotype**	**Source**
BY4743	*MAT***a**/α *his3*Δ*1*/*his3*Δ*1 leu2*Δ*0*/*leu2*Δ*0 LYS2*/*lys2*Δ*0 MET15*/*met15*Δ*0 ura3*Δ*0*/*ura3*Δ*0*	Invitrogen
BY4743 *cox23*Δ	*MAT***a**/α *his3*Δ*1*/*his3*Δ*1 leu2*Δ*0*/*leu2*Δ*0 LYS2*/*lys2*Δ*0 MET15*/*met15*Δ*0 ura3*Δ*0*/*ura3*Δ*0 *Δ*cox23*::*kanMX4*	Invitrogen
BY4741	*MAT***a ***his3*Δ*1 leu2*Δ*0 met15*Δ*0 ura3*Δ*0*	Invitrogen
BY4741 *cox23*Δ	*MAT***a ***his3*Δ*1 leu2*Δ*0 met15*Δ*0 ura3*Δ*0 *Δ*cox23*::*kanMX4*	Invitrogen
BY4741 *cox19*Δ	*MAT***a ***his3*Δ*1 leu2*Δ*0 met15*Δ*0 ura3*Δ*0 *Δ*cox19*::*kanMX4*	Invitrogen
BY4741 *cox17*Δ	*MAT***a ***his3*Δ*1 leu2*Δ*0 met15*Δ*0 ura3*Δ*0 *Δ*cox17*::*kanMX4*	Invitrogen
BY4741 *coa1*Δ	*MAT***a ***his3*Δ*1 leu2*Δ*0 met15*Δ*0 ura3*Δ*0 *Δ*coa1*::*kanMX4*	Invitrogen
BY4741 *coa2*Δ	*MAT***a ***his3*Δ*1 leu2*Δ*0 met15*Δ*0 ura3*Δ*0 *Δ*coa2*::*kanMX4*	Invitrogen
BY4741 *shy1*Δ	*MAT***a ***his3*Δ*1 leu2*Δ*0 met15*Δ*0 ura3*Δ*0 *Δ*shy1*::*kanMX4*	Invitrogen

### Mitochondrial Purification

Intact mitochondria were isolated from yeast as described previously 34. Total mitochondrial protein concentration was determined using Coomassie Plus Protein Assay Reagent (ThermoScientific). 

### Blue Native PAGE

Blue Native PAGE (BN-PAGE) was performed as previously described 35. Briefly, 20 to 30 μg isolated mitochondria was solubilized in sample buffer (1% digitonin, 0.5 M 6-aminocaproic acid, pH 7.0), incubated in ice for 20 min and then centrifuged (20,000 x* g* for 10 min at 4°C). Supernatants were mixed with 0.5 μl 5% Coomassie brilliant blue G250 and loaded on a NativePAGE Novex^TM^ 3-12% gradient polyacrylamide gel (Invitrogen) alongside a high-mass protein marker (GE Healthcare). 

### Immunoblotting

BN-PAGE mass-resolved complexes were detected after transfer to a polyvinylidene difluoride (PVDF) membrane. Alternatively, mitochondrial proteins were detected after separation of 10 to 30 μg solubilized and reduced mitochondria on 12% SDS-PAGE gel and transfer to nitrocellulose. Proteins were visualized using Supersignal (ThermoScientific) to detect horseradish peroxidase-conjugated secondary antibodies. Primary antibodies used were either purchased or generous gifts: anti-Cox1 and anti-Cox2 (Mitoscience), anti-Porin (Molecular Probes), Anti-Sdh2 (21st Century Biochemicals), anti-F1 ATPase (Dr. A. Tzagoloff), anti-Cyt1 and anti-Cox5a (Dr. B. Meunier), and anti-Cox13 (Dr. P. Rehling).

### Miscellaneous Assays

CcO activity in isolated mitochondria were determined spectrophotometrically by supplying reduced cytochrome* c* and following the initial rate of cytochrome *c* oxidation at 550 nm using an Agilent 8453 spectrophotometer. Reduced cytochrome *c* was prepared by adding equimolar amount of sodium hydrosulfite (Aldrich) to horse heart cytochrome* c* (Sigma) and desalting using a PD-10 gravity flow colum (GE Healthcare). The rate of oxygen consumption of cells grown in YP-Galactose (YPGal) media then carbon-swapped to YP-Glycerol/Lactate (YPGL) was determined from the linear response on a 5300A biological oxygen monitor (Yellow Springs Instruments Co.). Optical absorption spectroscopy was used to monitor mitochondrial heme pools. Two mg of purified mitochondria was suspended in 250 μl of distilled water. Same volume of a stock solution (200 mM NaOH, 40% pyridine) and 1.5 μl of 0.1 M K_3_Fe(CN)_6 _were added to the mitochondrial suspension. Each spectrum represents the calculated difference spectrum of the reduced (dithionite) minus oxidized (ferricyanide) cytochromes and was recorded by an Agilent 8453 spectrophotometer. Absorption maxima at 550 and 558 nm correspond to cytochromes *b*/*c* and *a*/*a*_3_, respectively.

For *in vivo* mitochondrial translation assay, cells were grown overnight in YPGal media to an OD_600_ of 1. [^35^S]-methionine labeling and sample preparation for 15% SDS-PAGE was performed as previously described 36. Gels were dried and radiolabelled mitochondrial proteins were visualized by overnight film autoradiography. Growth tests to determine the respiratory competency of yeast strains were performed on agar plates containing 2% glucose or 2% glycerol-2% lactate. Yeast cells were grown overnight in YPD medium and adjusted to an optical density at 600 nm of 0.5. Serial dilutions were spotted onto plates and incubated at 30°C for 2 days (glucose plates) or 4 to 8 days (glycerol-lactate plates). Bathocuproine sulfonate (BCS) and bathophenanthroline sulfonate (BPS) were purchased from Sigma.
